# Transparent TiO_2_ nanotube array photoelectrodes prepared via two-step anodization

**DOI:** 10.1186/s40580-014-0009-3

**Published:** 2014-04-04

**Authors:** Jin Young Kim, Kai Zhu, Nathan R Neale, Arthur J Frank

**Affiliations:** 1 Photo-electronic Hybrids Research Center, Korea Institute of Science and Technology (KIST), Seoul, 136-791 Korea; 2 Green Green School, Korea University, Seoul, 136-701 Korea; 3 National Renewable Energy Laboratory, Golden, 80401-3393 Colorado, USA

**Keywords:** Anodization Process, Transparent Conducting Oxide, Anodization Time, Step Anodization, Anodization Rate

## Abstract

Two-step anodization of transparent TiO_2_ nanotube arrays has been demonstrated with aid of a Nb-doped TiO_2_ buffer layer deposited between the Ti layer and TCO substrate. Enhanced physical adhesion and electrochemical stability provided by the buffer layer has been found to be important for successful implementation of the two-step anodization process. With the proposed approach, the morphology and thickness of NT arrays could be controlled very precisely, which in turn, influenced their optical and photoelectrochemical properties.

**Electronic supplementary material:**

The online version of this article (doi:10.1186/s40580-014-0009-3) contains supplementary material, which is available to authorized users.

## Correspondence/Findings

Aligned TiO_2_ nanotube (NT) arrays have attracted considerable interest owing to their versatility in a number of optoelectronic applications, such as solar cells [1–3], photocatalysis [4, 5], and electrochromics [6]. For instance, when used as electrodes in dye-sensitized solar cells (DSSCs), they were found to show higher light-harvesting and charge-collection efficiencies compared to their nanoparticle-based counterparts [7]. However, most anodized TiO_2_ NT arrays have been prepared using non-transparent Ti-foil substrates, limiting the light illumination geometry to just one side of the electrode. The limited illumination geometry lowers the performance of the optoelectronic devices because the incident light is attenuated by the device components (*e.g.* electrolyte) other than the TiO_2_ NT electrode [8]. Therefore, the benefits of TiO_2_ NT-based electrodes can only be fully utilized by using transparent TiO_2_ NT arrays. The challenges associated with preparing transparent TiO_2_ NT arrays have thus far limited the numbers of routes for fabricating these electrodes [9, 10]. Recently, we found that the transparent TiO_2_ NT array films could be prepared via a simple and reproducible approach involving the deposition of a conducting buffer layer between the Ti layer and transparent conducting oxide (TCO) substrate [11]. The buffer layer protects the TCO from degradation through a self-terminating mechanism that arrests the TiO_2_ NT growth on the Ti layer and improves the adhesion and electrical contact between the NTs and TCO.

A second challenge in TiO_2_ NT array synthesis has been the ability to precisely control the microstructure dimensions, extent of alignment, pore ordering, and degree of overlayer formation. These properties have a substantial impact on the device properties. For instance, we recently showed that removing the structural disorder of TiO_2_ NT arrays promoted more efficient charge transport in DSSCs [12]. Also, ordering of the NTs can affect their optical properties, in particular their ability to scatter light that affects the light-harvesting properties of the electrode. Strategies to control the NT dimensions and the extent of alignment have involved using different anodization potentials [13, 14], electrolytes [15, 16], drying conditions [12], and bath temperatures [4]. However, only a few studies have focused on the control of pore ordering [17] and overlayer formation [9, 18]. On the other hand, it is well known that highly ordered pore structures in alumina can be achieved via a two-step process [19, 20]. However, only a paucity of studies have investigated the use of the two-step anodization of TiO_2_ NT arrays [14, 17, 21], mostly owing to the absence of proper chemical etchants that dissolve only oxide NTs without dissolving the underlying substrate. Furthermore, all of these examples used non-transparent Ti foil as the substrate for growing NTs. For the reason given above, it would be highly useful to extend this two-step anodization technique to transparent TiO_2_ NT arrays.

The biggest challenge for the successful implementation of the two-step procedure for preparing transparent TiO_2_ NT arrays is overcoming the poor adhesion between the Ti layer and TCO substrate. During a typical two-step anodization, TiO_2_ NT arrays are grown to a certain thickness and then mechanically removed either by gas evolution [14] or by adhesive tapes [17]. We find that these approaches are not applicable to TiO_2_ NT arrays grown on TCO substrates. Removal of the initially grown NT layer with these approaches results in delamination of the remaining Ti film from the TCO substrate. This situation is largely attributed to the poor adhesion between the Ti film and the TCO substrate, unlike the case of NTs grown on Ti foil.

In this communication, we demonstrate the first two-step anodization procedure for growing transparent TiO_2_ NT array films on TCO substrates. Key to the synthesis is the aid of a buffer layer (*i.e.* Nb-doped TiO_2_; NTO), which is found to improve both the physical adhesion between the Ti layer and TCO and the electrochemical stability of the NT films during the anodization process. We also report on simple and versatile approaches to control the morphology of the NT arrays by using a post-growth pore widening procedure, which involves changing the anodization time.

**Figure 1:**
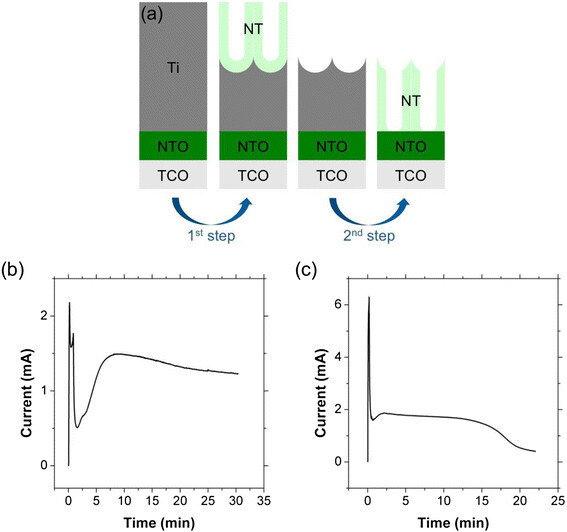
**(a) Schematic illustration of the two-step anodization process, and the anodization current profiles of (b) first step and (c) second step anodization.**

A schematic procedure for the two-step anodization of transparent TiO_2_ NT array films is illustrated in Figure 1a. The Ti thin film, which is deposited on the TCO substrate, is anodized to produce a sacrificial NT layer on top of the remaining Ti layer. Removal of this sacrificial layer leaves dimples on the Ti surface. These dimples serve as a template that directs pore formation during the second anodization step in which the remaining Ti is fully anodized. As described in the introduction, the strong adhesion of the Ti layer is very important for the selective removal of the sacrificial NT layer. In contrast to NT films grown directly on TCO, the presence of the NTO layer allows the first NT layer to be removed either by gas evolution or by the adhesive tape without delaminating the remaining Ti film. This indicates that the NTO layer improves the adhesion between the Ti film and TCO substrate. Figure 1b,c show typical anodization current profiles of the first and second stages of the two-step anodization process, respectively. It is interesting that the decreased current in the early stages of the anodization process recovers to steady state much faster in the second anodization. The current drop and subsequent recovery are reported to be related to the respective surface roughness of the Ti metal and higher degree of ordering in the final NT film during the second anodization [17]. The electrochemical stability of the NTO layer, which was reported in our previous study [11], is also observed in the anodization current profile of the second step (Figure 1c), where the anodization current does not increase even after complete anodization.

**Figure 2:**
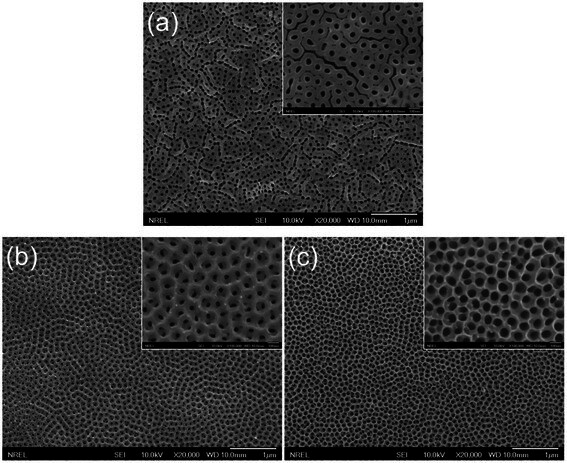
**Top-view SEM images of TiO**_**2**_ **NT arrays prepared by (a) single-step anodization process, (b) two-step anodization process, and (c) two-step anodization process and subsequent pore-widening process, where the inset in (a) shows the top surface morphology after Ar-ion etching and the insets in (b) and (c) show the images at the same magnification.**

The SEM images in Figure 2a display the surface morphology of a transparent TiO_2_ NT film prepared by the single-step anodization process. It can be seen that the film surface is covered by a porous overlayer and that the individual NTs are not very discernible. Formation of the porous overlayer [18] has been ascribed to the very low chemical etching rate of the oxide in the ethylene glycol-based electrolyte [9]. We find that the overlayer can be easily removed by ion-beam etching. The inset in Figure 2a shows the surface morphology of the NT film after ion-beam etching (1.2 keV, 26 mA, and 30 min). It is clear from the SEM image that the film is composed of densely packed NT arrays with little interstitial space between adjacent tubes. On the other hand, the surface morphology after the two-step anodization (Figure 2b) looks very similar to that prepared by the two-step anodization of Ti foil [17]. The surface morphology (Figure 2b) appears more ordered than that of the single-step counterpart (Figure 2a). Better ordering of pores after the two-step anodization can be ascribed to the smoother Ti surface after removing the sacrificial NT layer and the templating effect produced by the dimples. The average pore diameter measured from the magnified image (inset in Figure 2b) is 38 (± 7) nm, and the pore-to-pore distance is 117 (± 11) nm. The NT morphology can be further modified by the post-growth pore-widening process. Figure 2c shows that the average pore diameter of the NT film increases from 38 (± 7) nm (inset in Figure 2b) to 66 (± 5) nm after the pore widening, whereas the pore-to-pore distance essentially remains the same (112 ± 13 nm). This result indicates that the pore size can be controlled by varying the pore-widening time without disrupting the ordered arrangement of the NT pores. These observations suggest that the two-step anodization and subsequent pore widening are versatile ways to control the morphology of transparent TiO_2_ NT films.

**Figure 3:**
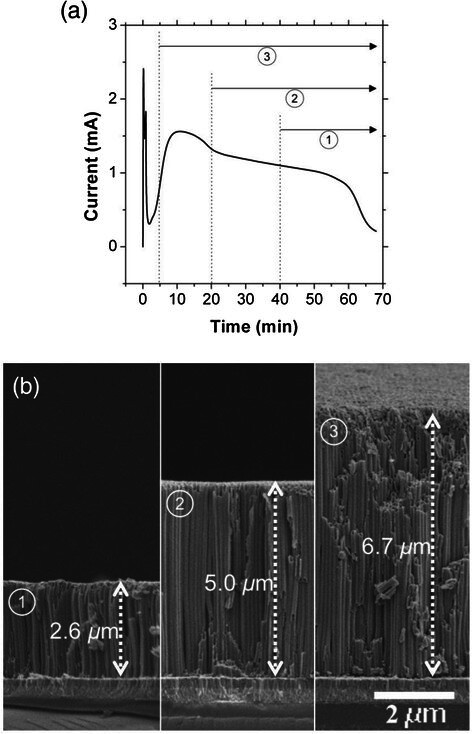
**(a) The strategy to control the thickness of the two-step anodized transparent TiO**_**2**_ **NT films, where the graph shows a typical anodization current profile of the single-step anodization and the circled numbers show the duration of the second step anodization, and (b) SEM images show NT films of various thicknesses; the circled numbers correspond to those in (a).**

Figure 3a shows a typical current profile of the single step anodization and the strategy to control the thickness of the transparent TiO_2_ NT films via the two-step anodization. To control the NT thickness, we changed the reaction time between the first and second anodization processes. The numbers in the circle indicate when the first step anodization is stopped and the second step anodization is started for three different NT samples. Figure 3b compares the cross-sectional SEM images of the transparent NT samples prepared by the two-step anodization processes described in Figure 3a after the subsequent pore widening process. It can be seen that the thickness of the NT films could be varied from 6.7 *μ* m to 2.6 *μ* m by changing the first anodization time from 5 min to 40 min. This is a very precise approach to control the NT thickness, which is easily adaptable to other anodic oxide systems, such as the porous alumina membrane. The approximate anodization times for the second step are 25 min, 45 min, and 50 min for samples 1, 2, and 3, respectively. It should be noted that the anodization rate of the second anodization process is higher than the first anodization, resulting in a shorter anodization time compared to that shown Figure 3a. The anodization rate for the second step is calculated to be 130 ~ 150 nm/min, assuming that the final 5 min does not contribute to the film growth, which is about twice as fast as the reported anodization rate for Ti foils [14]. The rate difference can be ascribed to the different properties of the starting Ti. Preliminary experiments show that the deposition temperature, which affects the crystallinity and preferred orientation of the Ti film, influences the anodization rate. It is noteworthy that the NT film thickness after the anodization process is significantly thicker than the starting Ti film (less than 2.6 *μ* m depending on the first anodization time), which is consistent with literature reports [9, 22]. Although the actual mechanism for the increased NT thickness is not clearly understood, it has been proposed that lateral motion of the anodized material into the walls of both the TiO_2_ NTs and anodized alumina structures is responsible for the NT film being thicker than the starting Ti film [23, 24]. Extremely small loss of Ti atoms owing to the re-attachment of the dissolved TiF_6_^2−^ and the subsequent oxide formation has also been proposed to account for the increased thickness of the relatively thin NT films [22].

**Figure 4:**
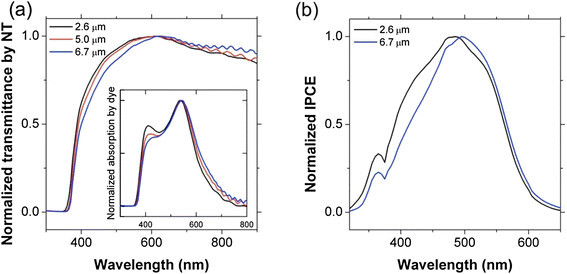
**(a) Normalized transmittance of transparent TiO**_**2**_ **NT arrays (without dye adsorption) with various thicknesses, where the inset shows the normalized absorption spectra only by the dye molecules adsorbed on the NT surfaces, and (b) Normalized IPCE spectra of DSSCs composed of transparent TiO**_**2**_ **NT arrays with different thicknesses.**

Figure 4a shows the normalized transmittance spectra of the transparent TiO_2_ NT films displayed in Figure 3b. The absolute value of the transmittance (not shown) decreased with increasing NT thickness, an observation which is consistent with a previous report [9]. In order to investigate the spectral response of the NT films, the transmittance spectra were normalized with respect to the maximum spectral values. The NT films have an optical band gap (~ 3.4 eV), which is slightly larger than that of bulk anatase TiO_2_ (~ 3.2 eV), regardless of the film thickness. Such a slight blue shift is attributed to the small crystallite size, which is limited by the wall thickness of the NTs [25]. Interestingly, the relative transmittance at shorter wavelengths (< 550 nm) decreases with increasing NT thickness, whereas the relative transmittance at longer wavelengths (> 550 nm) increases with increasing NT thickness. In other words, thicker NT films transmit less high-energy photons and more low-energy photons, implying that the light scattering at shorter wavelengths becomes more significant for thicker (at least up to 6.7 *μ* m) NT arrays. The inset in Figure 4a shows the normalized absorption spectra obtained by subtracting the raw transmittance spectra of bare TiO_2_ NT arrays from those of dye-adsorbed counterparts. The resulting spectra represent the absorption by just the adsorbed dye. The absorption by dye molecules exhibits a very similar behavior to the transmittance of the stained NT arrays. The amount of light absorption by dye molecules decreases with increasing NT thickness at shorter wavelengths, whereas it increases with increasing NT thickness at longer wavelengths. The higher transmittance of NT array electrodes at wavelengths > 550 nm should lead to better light absorption by dye molecules on the photoelectrodes, as more photons can reach the adsorbed dye molecules on the NT arrays due to the higher transmittance. As can be seen in Figure 4b, the thickness- and wavelength-dependent light absorption of the transparent TiO_2_ NT films, in turn, affects the properties of the NT-based DSSCs. The normalized IPCE spectra show that the photocurrent response follows the same behavior as the transmittance and net absorption spectra shown in Figure 4a. For instance, the largest difference between 2.6 *μ* m and 6.7 *μ* m samples appears at 410 nm in both the net absorption and IPCE spectra. The IPCE is normally approximated by the product of the following three factors: light-harvesting, charge-injection, and charge-collection efficiencies. Among these factors, the charge-injection efficiency is not influenced by the thickness of the NT arrays because the NT/dye interface is identical for all samples. Also, because with the NT length is much shorter than the electron diffusion length (~100 *μ* m) [26] the charge-collection efficiency of TiO_2_ NT-based DSSCs should be close to unity. Therefore, the IPCE spectra are influenced mainly by the light-harvesting efficiency that is represented by the amount of light absorption by the dye (*i.e.* the inset in Figure 4a). The advantage of having higher transmittance at wavelengths longer than 700 nm does not contribute substantially to the photoresponse for this particular DSSC system owing to the fact that the Z907 dye sensitizer absorbs very weakly at wavelengths above 700 nm. However, this effect will become more important for improving the performance of sensitized photoelectrodes incorporating active materials that absorb lower energy light, such as semiconductors (*i.e.* quantum dots) and IR-absorbing organic dyes.

In summary, we have demonstrated that the highly ordered transparent TiO_2_ NT arrays can be prepared by a two-step anodization with the aid of a Nb-doped TiO_2_ (NTO) buffer layer. Strong physical adhesion and electrochemical stability provided by the NTO layer are found to be crucial for the success of this approach. The morphology and thickness of the transparent TiO_2_ NT arrays can be controlled precisely by the post-growth pore widening and the anodization time between first and second step anodization. The controlled nanostructure, in turn, affects the optical properties of the transparent TiO_2_ NT arrays, which are shown to impact the photoelectrochemical properties of NT-based sensitized solar cells.

## Experimental section

### NT preparation

Transparent TiO_2_ NT arrays were prepared via an electrochemical anodization process [27]. Before depositing Ti metal films, 10 at% Nb-doped TiO_2_ (NTO) thin layers were deposited on the F-doped SnO_2_ (TEC15, Pilkington) substrates by RF-magnetron sputtering at 400°C using a ceramic target with the same composition under Ar flow (20 sccm) with a working pressure of 5 mTorr. Ti metal films with a thickness of 2.6 *μ* m were deposited on the NTO/TCO substrates using a Ti metal target (99.9%) under the same sputtering conditions. The Ti thin films were anodized in a two-electrode cell at 50 V (room temperature, Pt counter electrode) using 0.25 wt% NH_4_F (Aldrich, 99.9%) electrolyte in ethylene glycol (Aldrich, 99%) with 1 wt% of H_2_O. The anodization process was controlled by a source meter (Keithley, Model: 2425) connected to a computer. The applied voltage was increased with a ramp rate of 1 V/sec and 5 V/sec for first and second anodization processes, respectively [14]. After washing with water, the first NT layer was removed by applying a cathodic potential (−3 ~ −5 V) in 1 M H_2_SO_4_ aqueous solution [14] using a two-electrode cell with a platinum counter electrode, and then anodized again after washing with water and ethanol. The as-prepared samples were rinsed with water and ethanol, and dried with a gentle stream of N_2_. Then, for the pore widening, the as-anodized NT films were immersed in the formamide solution consisting of 0.15 M NH_4_F and 3.5 wt% H_2_O at 80°C for 5 min [11]. After rinsing and drying using the same conditions, the NT films were annealed at 400°C for 1 h under an ambient atmosphere.

### DSSC fabrication

For fabricating DSSCs, the films were stained with a 0.3 mM ethanolic solution of Z907 dye (cis-bis(isothiocyanato)(2,2′-bipyridyl-4,4′-dicarboxylato)(4,4′-di-nonyl-2′-bipyridyl)ruthenium(II)), and sandwich type devices were fabricated with Pt-loaded TCO substrates using the similar procedure described previously [11]. The electrolyte was composed of 1.0 M 1-methyl-3-propylimidazolium iodide, 30 mM I_2_, 0.5 M 1-butyl-1H-benzimidazole, and 0.1 M guanidinium thiocyanate in 3-methoxypropionitrile [28].

### Characterization

The microstructure and thickness of the NT arrays were characterized by field-emission scanning electron microscopy (FE-SEM). The optical absorption/transmission spectra and external quantum efficiencies were obtained using an IPCE measurement system (PV Measurements, Inc.).

## Authors’ original submitted files for images

Below are the links to the authors’ original submitted files for images. Authors’ original file for figure 1
Authors’ original file for figure 2
Authors’ original file for figure 3
Authors’ original file for figure 4
