# Supplementary figures and images for: Transparent TiO2 nanotube array photoelectrodes prepared via two-step anodization
Source: Nano Converg. 2014 Apr 4;1:9. doi: 10.1186/s40580-014-0009-3 (PMC5270970; doi:10.1186/s40580-014-0009-3)

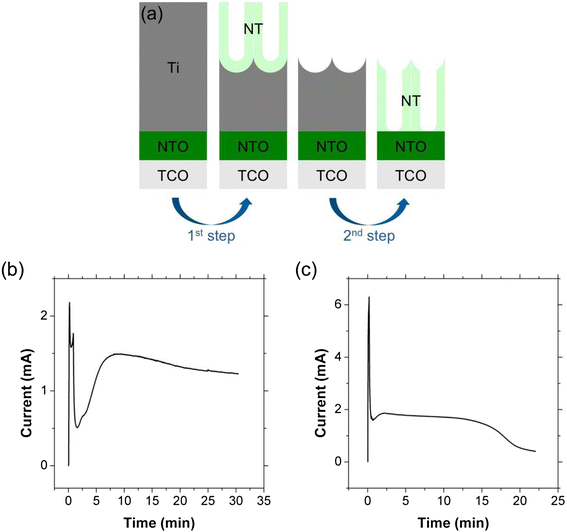

Supplement: Supplementary file 1 — Authors’ original file for figure 1 [file 40580_2014_9_MOESM1_ESM.gif]

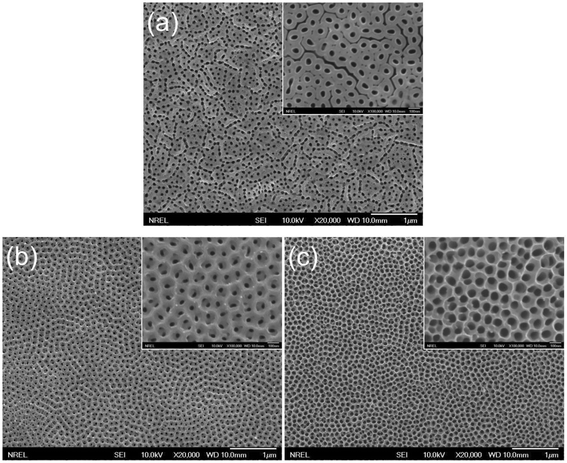

Supplement: Supplementary file 2 — Authors’ original file for figure 2 [file 40580_2014_9_MOESM2_ESM.gif]

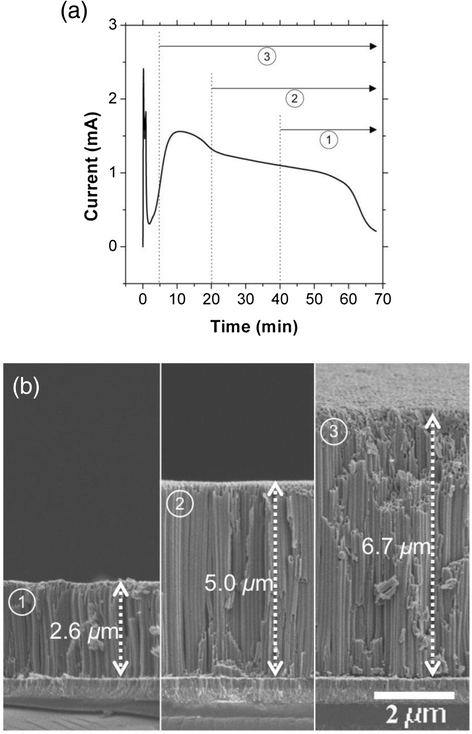

Supplement: Supplementary file 3 — Authors’ original file for figure 3 [file 40580_2014_9_MOESM3_ESM.gif]

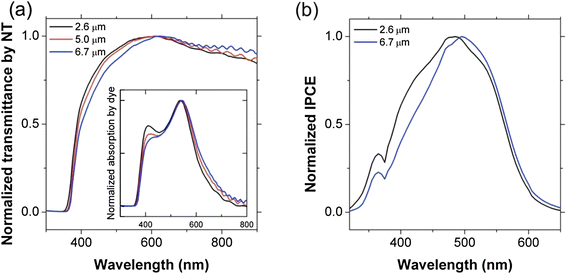

Supplement: Supplementary file 4 — Authors’ original file for figure 4 [file 40580_2014_9_MOESM4_ESM.gif]
